# Comparative effectiveness of TNF inhibitor vs IL-6 receptor inhibitor as monotherapy or combination therapy with methotrexate in biologic-experienced patients with rheumatoid arthritis: An analysis from the CorEvitas RA Registry

**DOI:** 10.1007/s10067-023-06588-7

**Published:** 2023-04-15

**Authors:** Anthony Sebba, Clifton O. Bingham, Vivian P. Bykerk, Stefano Fiore, Kerri Ford, Jud C. Janak, Dimitrios A. Pappas, Taylor Blachley, Swapna S. Dave, Joel M. Kremer, Miao Yu, Ernest Choy

**Affiliations:** 1 Rheumatology, Arthritis Associates, Palm Harbor, FL USA; 2 Division of Rheumatology, Johns Hopkins University, Baltimore, MD USA; 3 Inflammatory Arthritis Center, Hospital for Special Surgery, New York, NY USA; 4 Medical Affairs, Sanofi, Bridgewater, NJ USA; 5 Medical Affairs, Sanofi, Cambridge, MA USA; 6 CorEvitas, LLC, Waltham, MA USA; 7 Division of Rheumatology, Columbia University, New York, NY USA; 8 Department of Medicine, Center for Rheumatology, Albany Medical College, Albany, NY USA; 9 CREATE Centre, Division of Infection and Immunity, Cardiff University, Wales, UK

**Keywords:** Antirheumatic agents, Biological therapy, Interleukin-6 receptor inhibitors, Patient-reported outcomes, Rheumatoid arthritis, Tumor necrosis factor inhibitors

## Abstract

**Objective:**

Randomized controlled trials (RCTs) in biologic-naïve rheumatoid arthritis (RA) patients with high disease activity and inadequate response/intolerance to methotrexate have shown interleukin-6 (IL-6) receptor inhibitors (IL-6Ri) to be superior to tumor necrosis factor inhibitors (TNFi) as monotherapy. This observational study aimed to compare the effectiveness of TNFi vs IL-6Ri as mono- or combination therapy in biologic/targeted synthetic (b/ts) -experienced RA patients with moderate/high disease activity.

**Methods:**

Eligible b/ts-experienced patients from the CorEvitas RA registry were categorized as TNFi and IL-6Ri initiators, with subgroups initiating as mono- or combination therapy. Mixed-effects regression models evaluated the impact of treatment on Clinical Disease Activity Index (CDAI), patient-reported outcomes, and disproportionate pain (DP). Unadjusted and covariate-adjusted effects were reported.

**Results:**

Patients initiating IL-6Ri (*n* = 286) vs TNFi monotherapy (*n* = 737) were older, had a longer RA history and higher baseline CDAI, and were more likely to initiate as third-line therapy; IL-6Ri (*n* = 401) vs TNFi (*n* = 1315) combination therapy initiators had higher baseline CDAI and were more likely to initiate as third-line therapy. No significant differences were noted in the outcomes between TNFi and IL-6Ri initiators (as mono- or combination therapy).

**Conclusion:**

This observational study showed no significant differences in outcomes among b/ts-experienced TNFi vs IL-6Ri initiators, as either mono- or combination therapy. These findings were in contrast with the previous RCTs in biologic-naïve patients and could be explained by the differences in the patient characteristics included in this study. Further studies are needed to help understand the reasons for this discrepancy in the real-world b/ts-experienced population.

**Supplementary information:**

The online version contains supplementary material available at 10.1007/s10067-023-06588-7.

**Table:** 

**Key Points** *• Patients with rheumatoid arthritis (RA) often require switching between biologics or targeted synthetic (b/ts) disease-modifying anti-rheumatic drugs (DMARDs) to achieve their treatment target.* *• Head-to-head randomized controlled trials (RCTs) in biologic-naïve RA patients with high disease activity and inadequate response/intolerance to methotrexate have shown interleukin-6 receptor inhibitors (IL-6Ri) to be superior to tumor necrosis factor inhibitors (TNFi) as monotherapy; however, there are no RCTs comparing these therapies in a population previously treated with b/tsDMARDs (i.e., b/ts-experienced patients).* *• This observational study compared the effectiveness of TNFi vs IL-6Ri (as mono- or combination therapy) in b/ts-experienced RA patients with moderate or high disease activity and found no significant differences in clinical outcomes for the two treatments.* *• A discrepancy is noted between our study and RCTs, which have shown superiority of IL-6Ri therapy (albeit in biologic-naïve patients). Further analyses may help elucidate the reason for this discrepancy in the real-world b/ts-experienced population.*			

## Introduction

Rheumatoid arthritis (RA) is a global public health challenge, with increasing rates of age-standardized point prevalence and annual incidence [1]. If inadequately treated, RA may cause joint damage, disability, and other sequelae that impact the quality of life and lead to economic losses [2]. Early diagnosis and treatment are important to reduce this disease burden in patients with RA, and a treat-to-target (TTT) approach is recommended to achieve clinical remission or low disease activity (LDA). Over the years, this has become a realistic goal with the advent of effective medications, including conventional synthetic (cs), biologic (b), and targeted synthetic (ts) disease-modifying anti-rheumatic drugs (DMARDs) [3, 4].

At present, methotrexate (MTX), a csDMARD is considered an integral part of the first-line treatment strategy in patients with RA [3]. Further optimization of MTX dosage or addition of b/tsDMARDs is driven by the TTT approach. If a b/tsDMARD fails, switching to a b/tsDMARD of a different class is recommended to achieve the target; however, there are limited data to support the choice of drug class for this approach [3, 4].

Two of the currently approved b/tsDMARD therapy classes act via inhibition of tumor necrosis factor-alpha (TNF) or interleukin-6 receptor (IL-6R), thereby playing a vital role in RA via their anti-inflammatory effects [5, 6]. Although 10% to 50% of RA patients achieve remission in 6 to 12 months with the use of the first b/tsDMARD (such as TNF inhibitor [TNFi] or IL-6R inhibitor [IL-6Ri]), either as monotherapy or in combination with csDMARDs, a meaningful proportion of patients have active disease and progression of disability [7, 8]. Thus, patients require switching between b/tsDMARDs to achieve the target of remission [3, 4], and this decision can be informed by research on the comparative effectiveness of these therapies.

A randomized Phase 3 head-to-head (H2H) trial (MONARCH) in biologic-naïve RA patients, with high disease activity and intolerance or inadequate response to MTX, showed sarilumab (an IL-6Ri) monotherapy to be superior to adalimumab (a TNFi) monotherapy for reducing disease activity and signs/symptoms of RA [9]. Tocilizumab (another IL-6Ri) also demonstrated superior clinical response than adalimumab in a Phase 4 randomized controlled trial (RCT) as monotherapy in a similar population [10]. However, there is limited research comparing the relative effectiveness of TNFi vs IL-6Ri as monotherapy or in combination with csDMARDs in RA patients with moderate or high disease activity, who have previously been treated with b/tsDMARDs (i.e., b/ts-experienced patients), in a real-world patient population.

The CorEvitas RA registry (formerly known as Corrona) is a prospective, multicenter, real-world registry, launched in the United States (US), and collects data (at the time of a clinical encounter) of clinical outcomes and patient-reported outcomes (PROs) from both physicians and patients. At present, the registry includes information on > 56,000 patients with RA from 857 rheumatologists across 42 states in the US [11]. Based on the CorEvitas RA registry, a retrospective examination of prospectively collected data was conducted to assess the clinical outcomes in b/ts-experienced RA patients, who received TNFi or IL-6Ri as monotherapy or in combination with csDMARDs. The objective of the study was to compare the effectiveness of second- and third-line TNFi vs IL-6Ri (as mono- or combination therapy) in treating RA patients with moderate or high disease activity.

## Materials and methods

### Study design and patient population

This was a retrospective, observational study in which data from adult RA patients, within the US CorEvitas RA registry [11], were evaluated. The study was conducted in accordance with the Declaration of Helsinki, and all participating investigators obtained full ethics or institutional review board (IRB) approval (central IRB: New England Independent Review Board [NEIRB] number: 120160610 and/or individual approvals at sites). All registry patients were required to provide written informed consent prior to participation.

Adult patients with RA (≥ 18 years) who initiated a TNFi (adalimumab, certolizumab pegol, etanercept, golimumab, or infliximab) or IL-6Ri (sarilumab or tocilizumab) during or after January 2010 (until May 2020) were included. The study period was selected based on the approval and clinical availability of IL-6Ri and TNFi classes of therapeutics. Patients were included in the study if they had a history of one or two b/tsDMARDs prior to initiation, moderate (Clinical Disease Activity Index [CDAI]: 10 to ≤ 22) or high (CDAI: > 22) disease activity at initiation [12], and recorded a follow-up visit at 6 (± 3) months after therapy initiation. Patients who were not eligible to participate in the registry included those who: (i) had a diagnosis of juvenile idiopathic arthritis, psoriatic arthritis, spondylarthritis, ankylosing spondylitis, systemic lupus erythematosus, or any other form of autoimmune inflammatory arthritis; (ii) were only on csDMARDs; or (iii) were participating/planning to participate in any RA clinical trial.

### Study treatments

All RA patients with moderate or high disease activity were categorized as either TNFi or IL-6Ri initiators, both with subgroups initiating as monotherapy or combination therapy. Monotherapy initiators stopped the prior csDMARDs at the time of initiating TNFi or IL-6Ri or anytime earlier, and combination therapy initiators received MTX with or without other csDMARDs, in addition to TNFi or IL-6Ri.

### Study assessments

At the baseline visit (i.e., visit at which TNFi/IL-6Ri was started), the following variables were recorded for each patient: demographic characteristics, lifestyle status, history of comorbidities, medication use, and disease severity measures including the CDAI and PROs. Clinical outcomes collected were as follows:

#### Clinical disease activity index

Mean change and achievement of low disease activity (LDA; CDAI: ≤ 10); achievement of minimal clinically important difference (MCID, i.e., improvement by ≥ 6 [for moderate disease activity at baseline] or ≥ 12 [for high disease activity at baseline] units) in the CDAI from baseline to follow-up; and achievement of remission (CDAI: ≤ 2.8) [12, 13].

#### Patient-reported outcomes

Health Assessment Questionnaire-Disability Index (HAQ-DI) (mean change and achievement of improvement in the HAQ-DI of ≥ 0.22 or ≥ 0.30 units) [14, 15]; EuroQol-5 Dimension (EQ-5D) score (mean change); pain visual analog scale (VAS, 0–100) (mean change and achievement of improvement by ≥ 10 units); patient global assessment (VAS, 0–100) (mean change and achievement of improvement by ≥ 10 units) [16]; and fatigue (single-item VAS, 0–100) (mean change and achievement of improvement by ≥ 10 units) [17].

#### Disproportionate pain (DP) [18, 19]

Presence or absence of DP_1_ at 6 months among patients with DP_1_ at baseline; and presence or absence of DP_2_ at 6 months among patients with DP_2_ at baseline, for which:

Presence of DP_1_ is defined as:

$$\mathrm{TJC}-\mathrm{SJC }\ge 7$$

Presence of DP_2_ (among those with TJC > 0) is defined as:

$$\frac{\mathrm{SJC}}{\mathrm{TJC}}<0.5$$

where, TJC is 28-tender joint count and SJC is 28-swollen joint count.

The following outcomes were measured as exploratory analyses: response to prior TNFi therapies (using duration of previous TNFi) to investigate whether response/non-response to prior TNFi therapy channeled patients to different subsequent treatments (TNFi vs IL-6Ri). Also, change in the prednisone dose from baseline to 6 months (using baseline dose of prednisone) was evaluated for all patients.

No safety outcomes were assessed in the present study.

### Statistical analyses

Both TNFi and IL-6Ri initiators (as mono- or combination therapy) were compared at baseline and at the follow-up visit. Descriptive statistics were measured for each variable at baseline. Continuous variables were summarized using mean and standard deviation (SD), while categorical variables were reported as total number and proportion of each category. Univariate comparisons between therapy groups were performed using t-tests for continuous variables and chi-square tests for categorical variables.

Since the same patient can potentially contribute to multiple observations for repeated initiations within the same drug class or across different drug classes, mixed-effects regression models with random intercept for patient were used to account for the potential correlation among separate observations from the same patient.

For mean change in outcomes, the difference from baseline to six-month follow-up was calculated for each patient and used as the dependent variable in mixed-effect linear regression models. For binary outcomes, an indicator variable was created, measuring whether a patient achieved the outcome from baseline to follow-up or not. These indicator variables were then used as dependent variables in mixed-effect logistic regression models to predict the achievement of each outcome. Unadjusted and covariate-adjusted effects (mean change in effect [β, 95% confidence interval {CI}] for linear regressions and odds ratio [OR, 95% CI] for logistic regressions) were reported. The independent variables in all models included treatment group (TNFi as reference), the baseline value of the outcome variable, and a set of additional covariates (confounders) determined a priori to be likely to influence the outcome measures. Covariates also included those characteristics which were found to be significantly different at the baseline; For monotherapy initiators, covariates included biologic line of therapy, age, duration of RA, gender, work status, history of cardiovascular disease (CVD), CDAI, and morning stiffness; for combination therapy initiators, these were biologic line of therapy, history of CVD, CDAI, patient reported pain, prior use of csDMARDs, and opioids use. These analyses were replicated for monotherapy and combination therapy initiators.

Differences in duration of prior exposure to TNFi were also investigated among patients who had received TNFi earlier. Duration of prior TNFi therapy was used as a proxy for primary and secondary non-response, which may be associated with the effectiveness of subsequent TNFi [20, 21]. The last prior TNFi was identified among patients with a history of at least one prior TNFi, and the proportion of the population was reported with the available information as well as the mean (SD) and median (25^th^ percentile and 75^th^ percentile) duration of therapy. Also, the proportions of the population that persisted on therapy for at least 6 and 12 months were reported. This information was presented for all eligible initiators and by line of therapy. It was assumed that therapy discontinued prior to 6 months would be more likely to be associated with primary non-response.

Lastly, prednisone use was categorized as no use, dose < 10 mg, and dose ≥ 10 mg, and summarized at baseline and at 6 months for TNFi and IL-6Ri monotherapy and combination therapy initiators.

Sensitivity analyses were conducted for mean change in outcomes and prednisone use, where outcomes were reanalyzed by considering binary response outcomes as “non-responders” and imputing continuous outcomes with last observation carried forward (LOCF) for patients who discontinued a biologic prior to the six-months follow-up.

All analyses were performed using Stata 15 and/or SAS 9.4.

## Results

### Patient disposition and baseline characteristics

**Fig. 1:**
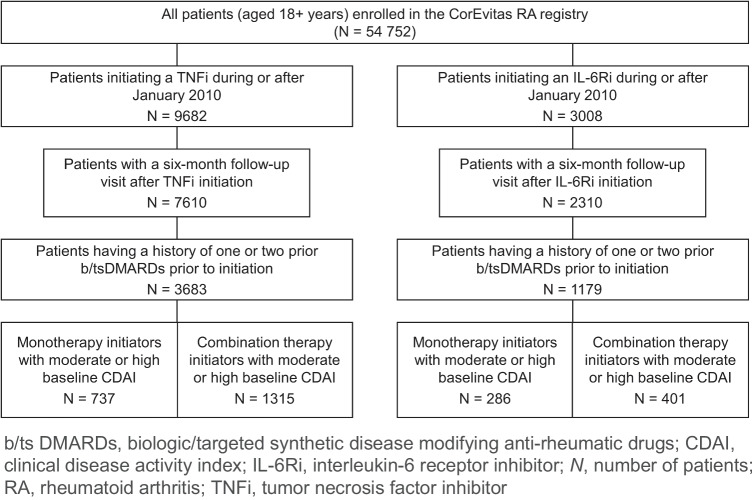
Patient disposition

Out of 9682 patients (with moderate or high disease activity) initiating TNFi, 737 and 1315 patients were included in the study as monotherapy and combination therapy initiators, respectively. Similarly, out of 3008 patients (with moderate or high disease activity) initiating IL-6Ri, 286 and 401 patients were included in the study as monotherapy and combination therapy initiators, respectively (Fig. 1).

Patients initiating IL-6Ri (*n* = 286) vs TNFi monotherapy (*n* = 737) were older (60.0 vs 55.4 years; *P* < 0.001), had a longer history of RA (12.2 vs 10.0 years; *P* = 0.001), higher CDAI at baseline (26.9 vs 24.9; *P* = 0.02), and were more likely to initiate as third-line therapy (57.0% vs 30.9%; *P* < 0.001). Further, patients initiating IL-6Ri (*n* = 401) vs TNFi (*n* = 1315) combination therapy had higher CDAI at baseline (26.7 vs 24.8; *P* = 0.007) and were more likely to initiate as third-line therapy (56.4% vs 28.7%; *P* < 0.001). The detailed baseline demographic and clinical characteristics are described in Table 1 and Table 2, respectively.

**Table 1:** Baseline demographic characteristics in monotherapy and combination therapy initiators by therapy class

Characteristics^a^	Monotherapy initiators			Combination therapy initiators		
	TNFi	IL-6Ri	*P*-value	TNFi	IL-6Ri	*P*-value^b^
Total, *N*	737	286		1315	401	
Age, years	55.4 (13.6)	60.0 (12.9)	< 0.001	57.5 (13.2)	58.1 (13.4)	0.42
Duration of RA, years	10.0 (9.7)	12.2 (10.1)	0.001	10.1 (9.2)	9.9 (9.3)	0.74
Female, *n* (%)	613 (83.2)	220 (76.9)	0.02	1055 (80.2)	305 (76.1)	0.07
Race (White), *n* (%)	616 (83.7)	231 (81.3)	0.37	1077 (82.3)	314 (78.7)	0.10
Smoking status, *n* (%)			0.45			0.32
Never	354 (48.2)	129 (45.6)		665 (51.1)	192 (48.2)	
Previous/Current	380 (51.8)	154 (54.4)		637 (48.9)	206 (51.8)	
Alcohol use, *n* (%)^c^	299 (43.0)	116 (43.3)	0.93	517 (41.3)	183 (48.0)	0.02
BMI, kg/m^2^	30.1 (7.2)	29.3 (7.1)	0.13	31.2 (8.1)	30.8 (7.5)	0.33
BMI category, *n* (%)			0.32			0.98
Underweight (BMI < 18.5)	12 (1.6)	7 (2.4)		20 (1.5)	7 (1.8)	
Normal weight (18.5 ≤ BMI < 25)	172 (23.4)	80 (28.0)		277 (21.1)	83 (20.8)	
Overweight (25 ≤ BMI < 30)	227 (30.9)	78 (27.3)		382 (29.1)	115 (28.7)	
Obese (BMI ≥ 30)	323 (44.0)	121 (42.3)		632 (48.2)	195 (48.8)	
History of comorbidities, *n* (%)						
CVD^d^	71 (9.6)	42 (14.7)	0.02	145 (11.0)	72 (18.0)	< 0.001
Hypertension	210 (28.6)	99 (34.7)	0.05	420 (31.9)	132 (33.1)	0.67
Hyperlipidemia^c^	84 (12.4)	36 (13.5)	0.66	144 (12.3)	47 (12.4)	0.97
Malignancy^e^	46 (6.2)	17 (5.9)	0.86	86 (6.5)	21 (5.2)	0.34
Serious infections^f^	55 (7.5)	28 (9.8)	0.22	89 (6.8)	37 (9.2)	0.10
Diabetes	83 (11.3)	35 (12.3)	0.66	136 (10.3)	37 (9.3)	0.53
Depression	302 (41.0)	122 (42.7)	0.62	524 (39.8)	150 (37.4)	0.38
Fibromyalgia^c^	71 (10.5)	18 (6.7)	0.08	71 (6.1)	24 (6.3)	0.86

^a^Values are mean (standard deviation) unless indicated otherwise

^b^*P*-values from unadjusted comparison tests of characteristic distributions between therapy groups

^c^Variables (for monotherapy initiators) with more than 5% of missing data

^d^History of CVD includes myocardial infarction, stroke, acute coronary syndrome, coronary artery disease, congestive heart failure, revascularization procedure including percutaneous coronary intervention, coronary artery bypass grafting or coronary artery stents, ventricular arrhythmia, cardiac arrest, unstable angina, peripheral arterial disease, other CVDs, pulmonary embolism, carotid artery disease, deep vein thrombosis, and transient ischemic attack

^e^History of malignancy includes lymphoma, lung cancer, breast cancer, non-melanoma skin cancer, and other cancer

^f^Serious infections include infections that led to hospitalization or intravenous antibiotics: joint/bursa, cellulitis, sinusitis, diverticulitis, sepsis, pneumonia, bronchitis, gastroenteritis, meningitis, urinary tract infection, upper respiratory tract infection, or infection of other specified sites

BMI, body mass index; CVD, cardiovascular disease; IL-6Ri, interleukin-6 receptor inhibitor; *n/N*, number of patients; RA, rheumatoid arthritis; SD, standard deviation; TNFi, tumor necrosis factor inhibitor

**Table 2:** Baseline clinical characteristics in monotherapy and combination therapy initiators by therapy class

Characteristics^a^	Monotherapy initiators			Combination therapy initiators		
	TNFi	IL-6Ri	*P*-value	TNFi	IL-6Ri	*P*-value^b^
Total, *N*	737	286		1315	401	
CDAI	24.9 (12.3)	26.9 (12.4)	0.02	24.8 (12.2)	26.7 (12.2)	0.007
Tender joint count	9.0 (7.1)	9.6 (7.2)	0.23	9.0 (7.0)	10.0 (7.4)	0.01
Swollen joint count	6.0 (5.3)	6.9 (5.3)	0.02	6.6 (5.3)	6.7 (5.4)	0.60
Physician-reported global assessment	42.8 (21.6)	45.5 (20.6)	0.06	40.8 (20.5)	44.3 (20.0)	0.003
Patient-reported global assessment	56.5 (23.4)	58.4 (24.2)	0.26	52.2 (24.7)	55.5 (23.5)	0.02
HAQ-DI^c^	1.1 (0.7)	1.3 (0.7)	< 0.001	1.1 (0.7)	1.2 (0.7)	0.08
EQ-5D^c^	0.7 (0.2)	0.6 (0.2)	0.20	0.7 (0.2)	0.7 (0.2)	0.15
Patient reported pain	59.8 (25.0)	62.2 (25.1)	0.18	54.3 (25.9)	57.3 (24.1)	0.04
Patient reported fatigue^c^	58.2 (27.3)	58.5 (27.7)	0.85	53.9 (27.7)	57.3 (26.9)	0.03
Morning stiffness, n (%)	678 (92.4)	274 (96.1)	0.03	1208 (92.4)	376 (94.5)	0.15
Morning stiffness duration, h^c,d^	2.3 (3.8)	2.4 (3.7)	0.64	2.2 (3.9)	2.3 (4.2)	0.66
DP_1_, *n* (%)^e^	165 (22.4)	65 (22.7)	0.91	260 (19.8)	87 (21.7)	0.40
DP_2_, *n* (%)^c,e^	228 (33.1)	82 (30.4)	0.41	356 (28.5)	119 (31.3)	0.28
Prior use of csDMARDs, *n* (%)			0.65			0.03
0	90 (12.2)	29 (10.1)		-	-	
1	275 (37.3)	109 (38.1)		642 (48.8)	221 (55.1)	
2 +	372 (50.5)	148 (51.7)		673 (51.2)	180 (44.9)	
Prior use of TNFi, *n* (%)			< 0.001			< 0.001
0	69 (9.4)	32 (11.2)		104 (7.9)	34 (8.5)	
1	540 (73.3)	177 (61.9)		984 (74.8)	254 (63.3)	
2	128 (17.4)	77 (26.9)		227 (17.3)	113 (28.2)	
Prior use of any non-TNFi, *n* (%)	123 (16.7)	99 (34.6)	< 0.001	202 (15.4)	130 (32.4)	< 0.001
Prednisone use, *n* (%)			0.01			0.18
No use	517 (70.1)	190 (66.4)		928 (70.6)	261 (65.1)	
Current use, missing dose	6 (0.8)	1		21 (1.6)	6 (1.5)	
Current use, dose < 10 mg	147 (19.9)	49 (17.1)		259 (19.7)	92 (22.9)	
Current use, dose ≥ 10 mg	67 (9.1)	46 (16.1)		107 (8.1)	42 (10.5)	
b/tsDMARD line of therapy, *n* (%)			< 0.001			< 0.001
Second	509 (69.1)	123 (43.0)		937 (71.3)	175 (43.6)	
Third	228 (30.9)	163 (57.0)		378 (28.7)	226 (56.4)	

^a^Values are mean (standard deviation) unless indicated otherwise

^b^*P*-values from unadjusted comparison tests of characteristic distributions between therapy groups

^c^Variables (for monotherapy initiators) with more than 5% of missing data

^d^Only calculated for those reporting morning stiffness

^e^DP_1_: tender joint count (TJC, 28) – swollen joint count (SJC, 28) ≥ 7; DP_2_: SJC (28)/TJC (28) < 0.5

b/tsDMARD, biologic/targeted synthetic disease modifying anti-rheumatic drug; CDAI, clinical disease activity index; csDMARDs, conventional synthetic disease modifying anti-rheumatic drugs; DP, disproportionate pain; HAQ-DI, Health Assessment Questionnaire-Disability Index; IL-6Ri, interleukin-6 receptor inhibitor; *n/N*, number of patients; SJC, swollen joint counts; TJC, tender joint count; TNFi, tumor necrosis factor inhibitor

### Outcome assessments

In unadjusted as well as adjusted analyses, no clinically or statistically significant differences were noted for disease activity measures, PROs, and DP between TNFi and IL-6Ri initiators, both as mono- or combination therapy, although there was one exception. In the unadjusted analyses of DP_1_ (all initiators) among the combination therapy group, higher odds of DP_1_ presence were noted at 6 months for IL-6Ri when compared with TNFi (17.8% vs 12.6%; OR: 1.64 [1.10, 2.45]; *P* = 0.015); however, this difference was not seen in the adjusted analyses. One-third of the TNFi and IL-6Ri monotherapy (37.0% vs 32.7%; adjusted OR [aOR]: 0.99 [0.59, 1.67]; Table 3) and combination therapy initiators (36.7% vs 31.2%; aOR: 0.96 [0.66, 1.38]; Table 4) achieved LDA.

**Table 3:** Results from mixed models evaluating the impact of treatment class on disease burden, disproportionate pain, and disease activity among monotherapy initiators

Outcomes	Six-month mean (SD)/response rate		Unadjusted^a^			Adjusted^b^		
	TNFi	IL-6Ri	β^c^	OR^c^	95% CI	β^c^	OR^c^	95% CI
Disease activity								
CDAI	17.9 (13.4)	19.0 (13.9)	0.42	−	-1.24, 2.09	0.20	−	-1.54, 1.93
Achievement of LDA	270/729 (37.0%)	92/281 (32.7%)	−	0.80	0.48, 1.33	−	0.99	0.59, 1.67
Achievement of remission	52/729 (7.1%)	20/281 (7.1%)	−	1.74	0.25, 11.90	−	1.86	0.23, 15.05
Achievement of MCID in CDAI	326/729 (44.7%)	127/281 (45.2%)	−	0.94	0.61, 1.46	−	1.06	0.67, 1.69
Disease burden								
HAQ-DI	1.0 (0.7)	1.2 (0.7)	0.02	−	-0.05, 0.09	0.01	−	-0.06, 0.08
HAQ-DI improvement ≥ 0.22	252/678 (37.2%)	110/267 (41.2%)	−	1.03	0.68, 1.56	−	1.13	0.72, 1.77
HAQ-DI improvement ≥ 0.30	192/678 (28.3%)	78/267 (29.2%)	−	0.81	0.49, 1.32	−	0.91	0.56, 1.47
Pain VAS	49.1 (28.5)	51.2 (28.5)	1.09	−	-2.40, 4.58	-0.03	−	-3.67, 3.61
Pain VAS improvement ≥ 10	348/735 (47.3%)	128/285 (44.9%)	−	0.75	0.47, 1.21	−	0.80	0.48, 1.35
Patient global assessment VAS	47.2 (26.7)	47.2 (27.2)	-0.60	−	-3.92, 2.71	-1.47	−	-4.91, 1.98
Patient global assessment VAS improvement ≥ 10	347/735 (47.2%)	146/285 (51.2%)	−	1.14	0.78, 1.69	−	1.24	0.82, 1.87
Fatigue VAS	51.4 (28.9)	53.2 (29.1)	1.06	−	-2.39, 4.52	0.85	−	-2.73, 4.42
Fatigue VAS improvement ≥ 10	287/674 (42.6%)	101/266 (38.0%)	−	0.73	0.45, 1.19	−	0.74	0.43, 1.27
EQ-5D	0.7 (0.2)	0.7 (0.2)	0.02	−	-0.01, 0.04	0.02	−	-0.01, 0.05
DP								
DP_1_: All initiators	120/731 (16.4%)	52/283 (18.4%)	−	1.56	0.39, 6.18	−	1.38	0.58, 3.26
DP_1_ at baseline, no DP_1_ at 6 months	92/162 (56.8%)	30/64 (46.9%)	−	0.49	0.17, 1.46	−	0.54	0.16, 1.83
DP_2_: All initiators	235/547 (43.0%)	86/213 (40.4%)	−	0.82	0.45, 1.51	−	0.87	0.46, 1.67
DP_2_ at baseline, no DP_2_ at 6 months	50/188 (26.6%)	16/69 (23.2%)	−	0.82	0.39, 1.73	−	0.65	0.19, 2.20

^a^Unadjusted models include treatment indicators and baseline value of outcome as independent variables

^b^Adjusted models include treatment indicators, baseline value of outcome, and covariates specified in the covariate list and those identified to be significantly different in baseline table (covariates of monotherapy initiators: biologic line of therapy, age, duration of RA, gender, work status, history of CVD, CDAI, and morning stiffness; covariates of combination therapy initiators: biologic line of therapy, history of CVD, CDAI, patient reported pain, prior use of csDMARDs, and opioids use.)

^c^Based on unadjusted and covariate-adjusted regression analyses (β [95% CI] for linear regressions and OR [95% CI] for logistic regressions) using TNFi group as the reference; β represents the expected difference in the mean change of outcomes from baseline to 6 months for IL-6Ri group compared to TNFi group

CDAI, clinical disease activity index; csDMARDs, conventional synthetic disease modifying anti-rheumatic drugs; CI, confidence interval; CVD, cardiovascular disease; DP, disproportionate pain; EQ-5D, EuroQol-5 Dimension score; HAQ-DI, Health Assessment Questionnaire-Disability Index; IL-6Ri, interleukin-6 receptor inhibitor; LDA, low disease activity; MCID, minimal clinically important difference; OR, odds ratio; RA, rheumatoid arthritis; SD, standard deviation; TNFi, tumor necrosis factor inhibitor; VAS, visual analog scale

**Table 4:** Results from mixed models evaluating the impact of treatment class on disease burden, disproportionate pain, and disease activity among combination therapy initiators

Outcomes	Six-month mean (SD)/response rate		Unadjusted^a^			Adjusted^b^		
	TNFi	IL-6Ri	β^c^	OR^c^	95% CI	β^c^	OR^c^	95% CI
Disease activity								
CDAI	16.7 (12.3)	18.7 (13.6)	1.12	−	-0.17, 2.40	0.48	−	-0.84, 1.81
Achievement of LDA	478/1301 (36.7%)	124/397 (31.2%)	−	0.81	0.56, 1.18	−	0.96	0.66, 1.38
Achievement of remission	114/1301 (8.8%)	31/397 (7.8%)	−	1.43	0.42, 4.86	−	1.23	0.34, 4.44
Achievement of MCID in CDAI	605/1301 (46.5%)	185/397 (46.6%)	−	0.89	0.66, 1.20	−	0.97	0.71, 1.30
Disease burden								
HAQ-DI	1.0 (0.7)	1.1 (0.7)	0.03	−	-0.02, 0.09	0.01	−	-0.05, 0.07
HAQ-DI improvement ≥ 0.22	456/1168 (39.0%)	142/382 (37.2%)	−	0.82	0.59, 1.15	−	0.89	0.63, 1.25
HAQ-DI improvement ≥ 0.30	348/1168 (29.8%)	111/382 (29.1%)	−	0.87	0.59, 1.28	−	0.98	0.66, 1.46
Pain VAS	45.3 (28.4)	47.8 (27.5)	0.97	−	-1.81, 3.76	0.21	−	-2.66, 3.08
Pain VAS improvement ≥ 10	611/1308 (46.7%)	181/401 (45.1%)	−	0.78	0.54, 1.11	−	0.82	0.56, 1.18
Patient global assessment VAS	42.0 (26.6)	45.5 (27.0)	1.73	−	-0.95, 4.41	1.30	−	-1.44, 4.04
Patient global assessment VAS improvement ≥ 10	656/1306 (50.2%)	191/400 (47.8%)	−	0.72	0.49, 1.05	−	0.74	0.51, 1.08
Fatigue VAS	46.4 (29.1)	49.3 (29.1)	1.18	−	-1.64, 3.99	1.13	−	-1.77, 4.03
Fatigue VAS improvement ≥ 10	509/1165 (43.7%)	175/379 (46.2%)	−	1.02	0.74, 1.41	−	0.99	0.71, 1.38
EQ-5D	0.7 (0.2)	0.7 (0.2)	-0.00	−	-0.02, 0.01	-0.00	−	-0.02, 0.02
DP								
DP_1_: All initiators	165/1310 (12.6%)	71/398 (17.8%)	−	1.64	1.10, 2.45	−	1.39	0.94, 2.06
DP_1_ at baseline, no DP_1_ at 6 months	160/258 (62.0%)	51/87 (58.6%)	−	0.83	0.42, 1.63	−	0.93	0.45, 1.89
DP_2_: All initiators	332/978 (33.9%)	115/311 (37.0%)	−	1.16	0.78, 1.73	−	1.06	0.70, 1.61
DP_2_ at baseline, no DP_2_ at 6 months	112/287 (39.0%)	38/100 (38.0%)	−	0.90	0.44, 1.88	−	1.00	0.47, 2.12

^a^Unadjusted models include treatment indicators and baseline value of outcome as independent variables

^b^Adjusted models include treatment indicators, baseline value of outcome, and covariates specified in the covariate list and those identified to be significantly different in baseline table (covariates of monotherapy initiators: biologic line of therapy, age, duration of RA, gender, work status, history of CVD, CDAI, and morning stiffness; covariates of combination therapy initiators: biologic line of therapy, history of CVD, CDAI, patient reported pain, prior use of csDMARDs, and opioids use.)

^c^Based on unadjusted and covariate-adjusted regression analyses (β [95% CI] for linear regressions and OR [95% CI] for logistic regressions) using TNFi group as the reference; β represents the expected difference in the mean change of outcomes from baseline to 6 months for IL-6i group compared to TNFi group

CDAI, clinical disease activity index; csDMARDs, conventional synthetic disease modifying anti-rheumatic drugs; CI, confidence interval; CVD, cardiovascular disease; DP, disproportionate pain; EQ-5D, EuroQol-5 Dimension score; HAQ-DI, Health Assessment Questionnaire-Disability Index; IL-6i, interleukin-6 receptor inhibitor; LDA, low disease activity; MCID, minimal clinically important difference; OR, odds ratio; RA, rheumatoid arthritis; SD, standard deviation; TNFi, tumor necrosis factor inhibitor; VAS, visual analog scale

In sensitivity analyses, no clinically meaningful differences were noted, with exception of the patient global assessment in the monotherapy initiators; IL-6Ri monotherapy initiators reported higher odds of achieving patient global assessment compared to TNFi monotherapy initiators (OR = 1.62; 1.12–2.35) (Online Supplementary Table 1 and Online Supplementary Table 2).

Among monotherapy (TNFi, *n* = 319; IL-6Ri, *n* = 115) and combination therapy (TNFi, *n* = 617; IL-6Ri, *n* = 173) initiators with available data on immediate prior TNFi therapy, no meaningful differences were noted between TNFi vs IL 6Ri for the duration of prior TNFi (Table 5).

**Table 5:** Duration of last prior TNFi, by mono/combination therapy and therapy class groups

	Monotherapy		Combination therapy	
	TNFi	IL-6Ri	TNFi	IL-6Ri
All initiators with history of at least one prior TNFi, *N*	668	254	1211	367
Available prior therapy duration, *N*	319	115	617	173
Duration (months)				
Mean (SD)	15.3 (22.0)	18.1 (24.4)	18.2 (27.7)	16.5 (22.1)
Median (P25, P75)	7 (3, 17)	10 (3.5, 21)	8 (3, 20)	10 (4, 18)
Duration ≥ 6 months, *n* (%)	174 (54.5)	72 (62.6)	370 (60.0)	116 (67.1)
Duration ≥ 12 months, *n* (%)	117 (36.7)	49 (42.6)	228 (37.0)	70 (40.5)
All second-line initiators, *N*	449	93	850	148
Available prior therapy duration, *N*	193	38	404	67
Duration (months)				
Mean (SD)	15.8 (23.5)	21.0 (27.2)	19.6 (29.8)	22.1 (26.8)
Median (P25, P75)	6 (3, 17)	11 (7, 22)	8 (3, 22)	12 (7, 25)
Duration ≥ 6 months, *n* (%)	101 (52.3)	29 (76.3)	244 (60.4)	53 (79.1)
Duration ≥ 12 months, *n* (%)	72 (37.3)	18 (47.4)	155 (38.4)	38 (56.7)
All third-line initiators, *N*	219	161	361	219
Available prior therapy duration, *N*	126	77	213	106
Duration (months)				
Mean (SD)	14.7 (19.6)	16.7 (23.0)	15.5 (23.2)	13.0 (17.8)
Median (P25, P75)	7 (3, 19)	8 (3, 21)	7 (3, 17.5)	7.5 (3, 15)
Duration ≥ 6 months, *n* (%)	73 (57.9)	43 (55.8)	126 (59.2)	63 (59.4)
Duration ≥ 12 months, *n* (%)	45 (35.7)	31 (40.3)	73 (34.3)	32 (30.2)

IL-6i, interleukin-6 receptor inhibitor; *n/N*, number of patients; P25, 25^th^ percentile; P75, 75^th^ percentile; SD, standard deviation; TNFi, tumor necrosis factor inhibitor

Further, no meaningful differences were found between TNFi and IL-6Ri initiators (as mono- or combination therapy) for the use of prednisone, with majority of the patients continuing either at their baseline dose or switching to a low dose/no use of prednisone after 6 months of treatment (Online Supplementary Table 3 and Online Supplementary Table 4).

## Discussion

In this retrospective real-world evaluation of a b/ts-experienced cohort, TNFi and IL-6Ri initiators had similar clinical outcomes (i.e., disease activity, PROs, and DP), regardless of whether they initiated as monotherapy or combination therapy.

Due to the limited number of real-world biologic-naïve patients initiating IL-6Ri monotherapy in this large retrospective registry, the present study did not evaluate biologic-naïve patients such as the ones included in the H2H trials, systematic literature reviews, and network meta-analysis, which have shown improved clinical outcomes with IL-6Ri when compared with TNFi as monotherapy [9, 10, 22–26]. Although evidence exists for similar clinical outcomes of TNFi and IL-6Ri as combination therapy in biologic-naïve patients [22, 23], no H2H trials in combination with a csDMARD have been conducted so far.

Compared with prior H2H trials [9, 10], patients in the present study were 4–6 years older, and had 2–4 years longer disease duration and half the disease activity (in terms of CDAI) at baseline. The H2H trials compared the efficacy of IL-6Ri with adalimumab in b/ts-naïve RA patients while the present study included the b/ts-experienced patients on IL-6Ri and various TNFi drugs (not limited to adalimumab). Further, the dose of IL-6Ri (tocilizumab) used in the trial [10] was higher as compared with the approved starting dose in clinical practice and the real-world studies, where tocilizumab (subcutaneous or intravenous) was initiated either at low doses or escalated over time as per the patient’s disease activity [27–29]. Lastly, the present study included those patients who either had prior use of csDMARDs and/or were on combination therapy with csDMARDs, while the patients in H2H trials were those considered inappropriate candidates for the continued treatment with MTX. All these differences with the populations included in the previous H2H trials may have contributed to a reduced difference in effectiveness between the two treatments in this study [30, 31].

In line with the recently published definition of “difficult-to-treat RA” by the European Alliance of Associations for Rheumatology (i.e., patients who have failed ≥ 2 b/tsDMARD therapies), there were approximately 30% TNFi and 57% IL-6Ri initiators in this study who would fall in this category and thus, would have been classified as refractory to the treatment [32]. Therefore, it is possible that the larger fraction of “difficult-to-treat patients” in the IL-6Ri cohort may have influenced the results in favor of the TNFi cohort. While most of the components included in the “difficult-to-treat RA” definition were adjusted in the present study, some of the factors (such as radiographic progression) were not adjusted.

There was a high proportion of patients who had prior TNFi exposure (88.8%–92.1%) in our study. Literature suggests that a better treatment response would occur when switching from a TNFi to an alternative mechanism of action therapy [33–36]. Since many patients initiating a TNFi had already failed another TNFi in our b/ts-experienced cohort, we investigated for a potential selection bias in patients who received a follow-on TNFi. Patients with secondary non-response to a TNFi may be more likely to respond to another TNFi than patients with primary non-response [20, 21]. Thus, duration of prior TNFi therapy was used as a proxy for primary and secondary non-response, assuming therapy discontinued within 6 months after initiation would be more likely to be associated with primary non-response. The distribution of prior TNFi discontinuation within or after 6 months of therapy did not differ between the TNFi and IL-6Ri cohorts. However, time on prior TNFi may not have been a good surrogate for primary and secondary non-response [36, 37].

The current analysis had some important differences compared with other studies, which had suggested better outcomes when patients were switched to a different class of biologics after a TNFi failure rather than rechallenged with another TNFi (i.e., cycling) [33–36]. In our study, CDAI was the primary outcome, whereas in some similar analyses, persistence was used as an outcome [33, 34, 36]; there were only 6 months of follow-up; and approximately one-third of the TNFi initiators and more than half of the IL-6Ri initiators were on their second treatment switch.

Though similar efficacy has been reported for TNFi and other biologics (with different mechanisms of action) in RA, there may be patient subsets with differences noted in clinical outcomes. For example, the AMPLE trial reported similar efficacy for abatacept and adalimumab in all patients with RA [38], while its exploratory analysis showed an association between seropositivity (anti-cyclic citrullinated peptide antibodies [ACPA] and/or rheumatoid factor) and better clinical response with abatacept than adalimumab [39]. Similarly, various subsets of RA patients have shown better treatment responses with IL-6Ri compared with TNFi [40–43]. Recently, machine learning was used to identify a rule to predict the treatment response to sarilumab and suggested that the subset of RA patients with ACPA and CRP > 12.3 mg/L might respond better to sarilumab than to adalimumab [40]; this finding was also validated in a real-world setting [41].

The present study was designed to better inform clinicians about treatment options in patients who have failed prior b/tsDMARDs. The major strengths of this study were its observational real-world nature (reflective of current clinical practices in the US) and the large number of enrolled patients [44]. Further, our methodology was based on logistic regression to control confounders, which is known to yield similar results as propensity score methods in observational studies [45, 46]. However, as with every retrospective observational study, there are limitations. Patients and physicians were unblinded to treatment, and there could be unidentified selection or channeling biases (such as factors affecting adherence to therapy, monitoring requirements, and beliefs and preferences of patients/physicians) that may influence the choice as well as outcomes of a therapy in clinical practice [37, 44]. Also, the visits occurred every 6 months in the study and thus, possible dose changes for the treatments that occurred in between visits might not have been captured accurately in the registry, especially if there were multiple changes. Clinical trials, on the other hand, frequently use pre-determined dose escalation schemas. This difference may have accounted for some of the discrepancy in the study findings. Further, there was no assessment done for the relationship between prednisone and/or non-prescription medications (such as non-steroidal anti-inflammatory drugs) with b/tsDMARDs, which was outside the scope of current analyses, and may have an impact on the effect of b/tsDMARD therapies. Lastly, the findings for DP_1_ and DP_2_ need to be validated in future studies.

In conclusion, no significant differences were noted in clinical outcomes for TNFi vs IL-6Ri initiators (as mono- or combination therapy) in b/ts-experienced patients in this observational study. Results from RCTs have shown that IL-6Ri therapy in biologic-naïve patients is more efficacious than TNFi therapy. This inconsistency may be explained by the fact that the present study included real-world b/ts-experienced patients. Further analyses may help understand the reasons for this inconsistency and optimize the clinical outcomes for patients with RA.

## Supplementary information

Below is the link to the electronic supplementary material.

Supplementary file1 (PDF 272 KB)

## Data Availability

Data are available from CorEvitas, LLC through a commercial subscription agreement and are not publicly available. No additional data are available from the authors.

## Declarations

### Previous publications

The data in part were presented at American College of Rheumatology (ACR) Convergence 2021 (November 01–10, 2021).

Reference: Sebba A, Bingham C, Bykerk V, et al. (2021) Comparative Effectiveness of TNF Inhibitor vs IL-6 Receptor Inhibitor as Monotherapy or Combination Therapy with Methotrexate in Patients with Rheumatoid Arthritis: Analysis from CorEvitas’ RA Registry [abstract]. Arthritis Rheumatol 73 (suppl 9). Accessed on: November 30, 2022.
