# Supplementary material for: Comparative effectiveness of TNF inhibitor vs IL-6 receptor inhibitor as monotherapy or combination therapy with methotrexate in biologic-experienced patients with rheumatoid arthritis: An analysis from the CorEvitas RA Registry
Source: Clin Rheumatol. 2023 Apr 15;42(8):2037–51. doi: 10.1007/s10067-023-06588-7 (PMC10345054; doi:10.1007/s10067-023-06588-7)
Supplement: Supplementary file 1 — Supplementary file1 (PDF 272 KB) [file 10067_2023_6588_MOESM1_ESM.pdf]

CLINICAL RHEUMATOLOGY

Comparative effectiveness of TNF inhibitor vs IL-6 receptor inhibitor as monotherapy or combination therapy with methotrexate in biologic-experienced patients with rheumatoid arthritis: An analysis from the CorEvitas RA Registry

Anthony Sebba (ID: 0000-0002-3028-9365),[1] Clifton O Bingham (ID: 0000-0002-4752-5029),[2] Vivian P Bykerk (ID: 0000-0002-1219-3845),[3] Stefano Fiore (ID: 0000-0002-3785-2448),[4] Kerri Ford,[5] Jud C Janak,[6*] Dimitrios A Pappas (ID: 0000-0001-8338-027X),[6,7] Taylor Blachley,[6*] Swapna S Dave,[6*] Joel M Kremer (ID: 0000-0001-6674-9901),[6,8] Miao Yu,[6] Ernest Choy (ID: 0000-0003-4459-8609)[9]

[1]Department of Rheumatology, University of South Florida, Tampa, Florida, United States of America

[2]Division of Rheumatology, Johns Hopkins University, Baltimore, Maryland, United States of America

[3]Inflammatory Arthritis Center, Hospital for Special Surgery, New York, NY, United States of America

[4]Medical Affairs, Sanofi, Bridgewater, New Jersey, United States of America

[5]Medical Affairs, Sanofi, Cambridge, Massachusetts, United States of America

[6]CorEvitas, LLC, Waltham, Massachusetts, United States of America

[7]Division of Rheumatology, Columbia University, New York, NY, United States of America

[8]Department of Medicine, Center for Rheumatology, Albany Medical College, Albany, New York, United States of America

[9]CREATE Centre, Division of Infection and Immunity, Cardiff University, Wales, United Kingdom

**Affiliation at the time of study.*

Corresponding author: Anthony Sebba; email ID: anthonysebba@gmail.com

SUPPLEMENTARY MATERIAL

Online Supplementary Table 1 Sensitivity analysis: Results from mixed models evaluating the impact of treatment class on disease burden, disproportionate pain, and disease activity among monotherapy initiators

Outcomes	Six-month mean (SD)/response rate		Unadjusted[a]			Adjusted[b]		
	TNFi	IL-6Ri	β[c]	OR[c]	95% CI	β[c]	OR[c]	95% CI
Disease activity								
CDAI	19.2 (13.1)	19.7 (13.6)	-0.64	–	-2.15, 0.87	-0.92	–	-2.51, 0.68
Achievement of LDA	200/732 (27.3%)	79/283 (27.9%)	–	1.15	0.75, 1.74	–	1.31	0.86, 2.00
Achievement of remission	42/732 (5.7%)	18/283 (6.4%)	–	2.12	0.26, 17.17	–	2.11	0.21, 20.71
Achievement of MCID in CDAI	248/732 (33.9%)	108/283 (38.2%)	–	1.19	0.81, 1.75	–	1.24	0.82, 1.86
Disease burden								
HAQ-DI	1.0 (0.7)	1.2 (0.7)	0.02	–	-0.04, 0.08	0.01	–	-0.06, 0.07
HAQ-DI improvement ≥0.22	195/678 (28.8%)	96/268 (35.8%)	–	1.31	0.89, 1.93	–	1.42	0.92, 2.21
HAQ-DI improvement ≥0.30	149/678 (22.0%)	67/268 (25.0%)	–	1.03	0.66, 1.60	–	1.12	0.71, 1.78
Pain VAS	51.4 (28.2)	51.9 (27.9)	-0.88	–	-4.01, 2.26	-1.88	–	-5.16, 1.40
Pain VAS improvement ≥10	256/735 (34.8%)	112/286 (39.2%)	–	1.18	0.83, 1.67	–	1.24	0.85, 1.82
Patient global assessment VAS	49.4 (26.3)	48.9 (26.6)	-1.33	–	-4.26, 1.59	-2.77	–	-5.83, 0.28
Patient global assessment VAS improvement ≥10	258/735 (35.1%)	125/286 (43.7%)	–	1.47	1.04, 2.09	–	1.62	1.12, 2.35
Fatigue VAS	53.4 (28.7)	54.2 (28.5)	0.79	–	-2.22, 3.81	0.44	–	-2.70, 3.58
Fatigue VAS improvement ≥10	208/677 (30.7%)	82/267 (30.7%)	–	0.98	0.69, 1.41	–	1.02	0.69, 1.50
EQ-5D	0.7 (0.2)	0.7 (0.2)	0.02	–	-0.01, 0.04	0.02	–	-0.00, 0.05
DP								
DP_1: All initiators	127/734 (17.3%)	52/283 (18.4%)	–	1.18	0.26, 5.47	–	1.25	0.44, 3.55
DP_1 at baseline, no DP_1 at 6 months	69/163 (42.3%)	26/64 (40.6%)	–	0.84	0.30, 2.39	–	0.98	0.35, 2.78
DP_2: All initiators	236/585 (40.3%)	84/221 (38.0%)	–	0.86	0.52, 1.42	–	0.91	0.52, 1.57
DP_2 at baseline, no DP_2 at 6 months	35/201 (17.4%)	11/71 (15.5%)	–	0.77	0.21, 2.82	–	0.41	0.08, 2.25

[a]Unadjusted models include treatment indicators and baseline value of outcome as independent variables. [b]Adjusted models include treatment indicators, baseline value of outcome, and covariates specified in the covariate list and those identified to be significantly different in baseline table (covariates of monotherapy initiators: biologic line of therapy, age, duration of RA, gender, work status, history of CVD, CDAI, and morning stiffness; covariates of combination therapy initiators: biologic line of therapy, history of CVD, CDAI, patient reported pain, prior use of csDMARDs, and opioids use.). [c]Based on unadjusted and covariate-adjusted regression analyses (β [95% CI] for linear regressions and OR [95% CI] for logistic regressions) using TNFi group as the reference; β represents the expected difference in the mean change of outcomes from baseline to 6 months for IL-6Ri group compared to TNFi group.
CDAI, clinical disease activity index; csDMARDs, conventional synthetic disease modifying anti-rheumatic drugs; CI, confidence interval; CVD, cardiovascular disease; DP, disproportionate pain; EQ-5D, EuroQol-5 Dimension score; HAQ-DI, Health Assessment Questionnaire-Disability Index; IL-6Ri, interleukin-6 receptor inhibitor; LDA, low disease activity; MCID, minimal clinically important difference; OR, odds ratio; RA, rheumatoid arthritis; SD, standard deviation; TNFi, tumor necrosis factor inhibitor; VAS, visual analog scale.

Online Supplementary Table 2 Sensitivity analysis: Results from mixed models evaluating the impact of treatment class on disease burden, disproportionate pain, and disease activity among combination therapy initiators

Outcomes	Six-month mean (SD)/response rate		Unadjusted[a]			Adjusted[b]		
	TNFi	IL-6Ri	β[c]	OR[c]	95% CI	β[c]	OR[c]	95% CI
Disease activity								
CDAI	17.9 (12.7)	19.5 (13.1)	0.60	−	-0.65, 1.84	0.03	−	-1.25, 1.31
Achievement of LDA	404/1303 (31.0%)	109/399 (27.3%)	−	0.89	0.68, 1.18	−	1.02	0.77, 1.35
Achievement of remission	95/1303 (7.3%)	29/399 (7.3%)	−	1.07	0.69, 1.65	−	1.33	0.84, 2.10
Achievement of MCID in CDAI	501/1303 (38.4%)	160/399 (40.1%)	−	1.00	0.79, 1.27	−	1.07	0.83, 1.37
Disease burden								
HAQ-DI	1.0 (0.7)	1.1 (0.7)	0.02	−	-0.03, 0.07	0.01	−	-0.04, 0.06
HAQ-DI improvement ≥0.22	367/1169 (31.4%)	117/382 (30.6%)	−	0.91	0.69, 1.20	−	0.95	0.72, 1.26
HAQ-DI improvement ≥0.30	274/1169 (23.4%)	91/382 (23.8%)	−	0.97	0.73, 1.30	−	1.07	0.80, 1.44
Pain VAS	46.2 (28.1)	49.1 (27.2)	1.15	−	-1.43, 3.72	0.50	−	-2.16, 3.16
Pain VAS improvement ≥10	524/1309 (40.0%)	152/401 (37.9%)	−	0.84	0.65, 1.09	−	0.88	0.68, 1.15
Patient global assessment VAS	43.6 (26.5)	46.1 (26.6)	0.58	−	-1.93, 3.08	0.17	−	-2.39, 2.73
Patient global assessment VAS improvement ≥10	544/1307 (41.6%)	167/401 (41.6%)	−	0.92	0.72, 1.18	−	0.97	0.75, 1.26
Fatigue VAS	48.0 (29.1)	49.1 (29.0)	-1.10	−	-3.70, 1.50	-1.26	−	-3.92, 1.40
Fatigue VAS improvement ≥10	405/1166 (34.7%)	147/380 (38.7%)	−	1.14	0.85, 1.52	−	1.16	0.85, 1.57
EQ-5D	0.7 (0.2)	0.7 (0.2)	0.00	−	-0.02, 0.02	0.00	−	-0.02, 0.02
DP								
DP_1: All initiators	174/1311 (13.3%)	74/399 (18.5%)	−	1.54	1.07, 2.22	−	1.34	0.93, 1.93
DP_1 at baseline, no DP_1 at 6 months	140/259 (54.1%)	41/87 (47.1%)	−	0.62	0.26, 1.46	−	0.73	0.32, 1.69
DP_2: All initiators	329/1021 (32.2%)	127/324 (39.2%)	−	1.37	0.99, 1.88	−	1.29	0.92, 1.80
DP_2 at baseline, no DP_2 at 6 months	91/297 (30.6%)	27/105 (25.7%)	−	0.69	0.31, 1.52	−	0.78	0.34, 1.80

[a]Unadjusted models include treatment indicators and baseline value of outcome as independent variables. [b]Adjusted models include treatment indicators, baseline value of outcome, and covariates specified in the covariate list and those identified to be significantly different in baseline table (covariates of monotherapy initiators: biologic line of therapy, age, duration of RA, gender, work status, history of CVD, CDAI, and morning stiffness; covariates of combination therapy initiators: biologic line of therapy, history of CVD, CDAI, patient reported pain, prior use of csDMARDs, and opioids use.). [c]Based on unadjusted and covariate-adjusted regression analyses (β [95% CI] for linear regressions and OR [95% CI] for logistic regressions) using TNFi group as the reference; β represents the expected difference in the mean change of outcomes from baseline to 6 months for IL-6i group compared to TNFi group.

CDAI, clinical disease activity index; csDMARDs, conventional synthetic disease modifying anti-rheumatic drugs; CI, confidence interval; CVD, cardiovascular disease; DP, disproportionate pain; EQ-5D, EuroQol-5 Dimension score; HAQ-DI, Health Assessment Questionnaire-Disability Index; IL-6i, interleukin-6 receptor inhibitor; LDA, low disease activity; MCID, minimal clinically important difference; OR, odds ratio; RA, rheumatoid arthritis; SD, standard deviation; TNFi, tumor necrosis factor inhibitor; VAS, visual analog scale.

Online Supplementary Table 3 Prednisone use category at six-month follow-up by prednisone use category at initiation

**Prednisone use at 6 months**	**Prednisone use at initiation, *N* (%)**[a]		
	**No use**	**Dose <10 mg**	**Dose ≥10 mg**
TNFi monotherapy initiators	515	147	67
No use	463 (90)	43 (29)	13 (19)
Dose <10 mg	40 (8)	100 (68)	14 (21)
Dose ≥10 mg	12 (2)	4 (3)	40 (60)
TNFi combination therapy initiators	920	258	106
No use	836 (91)	62 (24)	30 (28)
Dose <10 mg	61 (7)	187 (72)	22 (21)
Dose ≥10 mg	23 (3)	9 (3)	54 (51)
IL-6Ri monotherapy initiators	189	49	46
No use	170 (90)	11 (22)	10 (22)
Dose <10 mg	12 (6)	33 (67)	7 (15)
Dose ≥10 mg	7 (4)	5 (10)	29 (63)
IL-6Ri combination therapy initiators	258	92	42
No use	242 (94)	25 (27)	9 (21)
Dose <10 mg	11 (4)	63 (68)	7 (17)
Dose ≥10 mg	5 (2)	4 (4)	26 (62)

[a]Sample size for prednisone use calculation excludes those in "Current use, missing dose" category at baseline and/or six-month follow-up.

IL-6Ri, interleukin-6 receptor inhibitor; TNFi, tumor necrosis factor inhibitor.

Online Supplementary Table 4 Sensitivity analysis: Prednisone use category at six-month follow-up by prednisone use category at initiation

Prednisone use at 6 months	Prednisone use at initiation, *N* (%)		
	**No use**	**Dose <10 mg**	**Dose ≥10 mg**
TNFi monotherapy initiators	495	139	59
No use	463 (94)	43 (31)	13 (22)
Dose <10 mg	29 (6)	87 (63)	11 (19)
Dose ≥10 mg	3 (1)	9 (6)	35 (59)
TNFi combination therapy initiators	889	255	105
No use	836 (94)	62 (24)	30 (29)
Dose <10 mg	43 (5)	184 (72)	26 (25)
Dose ≥10 mg	10 (1)	9 (4)	49 (47)
IL-6Ri monotherapy initiators	186	48	45
No use	170 (91)	11 (23)	10 (22)
Dose <10 mg	11 (6)	32 (67)	6 (13)
Dose ≥10 mg	5 (3)	5 (10)	29 (64)
IL-6Ri combination therapy initiators	253	91	41
No use	242 (96)	25 (27)	9 (22)
Dose <10 mg	7 (3)	63 (69)	6 (15)
Dose ≥10 mg	4 (2)	3 (3)	26 (63)

IL-6Ri, interleukin-6 receptor inhibitor; TNFi, tumor necrosis factor inhibitor.
